# Lico A Enhances Nrf2-Mediated Defense Mechanisms against *t*-BHP-Induced Oxidative Stress and Cell Death via Akt and ERK Activation in RAW 264.7 Cells

**DOI:** 10.1155/2015/709845

**Published:** 2015-10-20

**Authors:** Hongming Lv, Hua Ren, Lidong Wang, Wei Chen, Xinxin Ci

**Affiliations:** ^1^Institute of Translational Medicine, Department of Ophthalmology, The First Hospital, Jilin University, Changchun 130001, China; ^2^Key Laboratory of Zoonosis Research, Ministry of Education, College of Animal Science and Veterinary Medicine, Jilin University, 5333 Xi'an Road, Changchun 130062, China

## Abstract

Licochalcone A (Lico A) exhibits various biological properties, including anti-inflammatory and antioxidant activities. In this study, we investigated the antioxidative potential and mechanisms of Lico A against *tert*-butyl hydroperoxide- (*t*-BHP-) induced oxidative damage in RAW 264.7 cells. Our results indicated that Lico A significantly inhibited *t*-BHP-induced cytotoxicity, apoptosis, and reactive oxygen species (ROS) generation and reduced glutathione (GSH) depletion but increased the glutamate-cysteine ligase modifier (GCLM) subunit and the glutamate-cysteine ligase catalytic (GCLC) subunit genes expression. Additionally, Lico A dramatically upregulated the antioxidant enzyme heme oxygenase 1 (HO-1) and nuclear factor erythroid 2-related factor 2 (Nrf2), which were associated with inducing Nrf2 nuclear translocation, decreasing Keap1 protein expression and increasing antioxidant response element (ARE) promoter activity. Lico A also obviously induced the activation of serine/threonine kinase (Akt) and extracellular signal-regulated kinase (ERK), but PI3K/Akt and ERK inhibitors treatment displayed clearly decreased levels of LicoA-induced Nrf2 nuclear translocation and HO-1 expression, respectively. Furthermore, Lico A treatment markedly attenuated *t*-BHP-induced oxidative damage, which was reduced by treatment with PI3K/Akt, ERK, and HO-1 inhibitors. Therefore, Lico A might have a protective role against *t*-BHP-induced cytotoxicity by modulating HO-1 and by scavenging ROS via the activation of the PI3K/Akt and ERK/Nrf2 signaling pathways.

## 1. Introduction

Various severe imbalances in the systems involved in generating and scavenging reactive oxygen species (ROS) could induce oxidative damage [1]. Moreover, oxidative stress plays a vital role in the mechanisms of various diseases, including aging, cancer, and inflammation [2]. Under oxidative stress conditions, various cells, including macrophages, have developed their own defensive mechanisms to counteract ROS generation via the induction of intracellular phase II enzymes, such as heme oxygenase-1 [3]. Increasing evidence has shown that natural products such as dietary phytochemicals exert protective effects by not only scavenging ROS but also inducing the de novo expression of antioxidant genes [4].

Reduced glutathione (GSH) is not only the most abundant thiol antioxidant in cells, but also a key intracellular antioxidant in mammals, which involves in scavenging free radicals, maintaining redox status, and inhibiting cell apoptosis and is regulated by glutamate-cysteine ligase (GCL) [5–7]. GCL, a rate-limiting enzyme of GSH biosynthesis, is a heterodimer which consisted of the glutamate-cysteine ligase modifier (GCLM) subunit and the glutamate-cysteine ligase catalytic (GCLC) subunit [8, 9]. In addition, heme oxygenase-1 (HO-1), which is a significant antioxidant gene, plays a crucial role in maintaining the cellular redox homeostasis against oxidative stress [10]. Previous reports have shown that HO-1 could catalyze the oxidative degradation of heme to biliverdin, which is then reduced to produce bilirubin, which is a potent antioxidant [11]. Importantly, GCLC, GCLM, and HO-1 genes expression are regulated by nuclear factor-erythroid 2 related factor 2 (Nrf2). Nrf2 plays a critical role in protecting cells against oxidative stress via regulating antioxidant responses [12, 13]. Under normal conditions, the inactive form of Nrf2 is bound to Keap1 (Kelch-like ECH-associated protein 1, also known as a repressor of Nrf2) in the cytoplasm. Under stress conditions, Nrf2 dissociates from Keap1, translocates into the nucleus, and binds to the antioxidant response element (ARE), which results in the expression of several antioxidant and detoxification genes, including HO-1 [14]. Interestingly, the mechanism by which Nrf2 is released from the Keap1-Nrf2 complex remains to be demonstrated. However, recently it is reported that several signal transduction pathways, which are protein kinase C (PKC) [15, 16], phosphatidylinositol 3-kinase (PI3K) [17], and mitogen-activated protein kinase (MAPK) pathways [18], may also play a significant role in involving in the regulation of Nrf2 nuclear translocation. Flavonoids, which are naturally occurring bioactive compounds, are extensively distributed in natural products such as vegetables, fruits, and many medicinal plants [19]. Flavonoids, particularly flavonols, possess a range of pharmaceutical activities, such as anti-inflammatory, antioxidant, hepatoprotective, antiviral, and anticarcinogenic activities [20, 21]. Licochalcone A (Lico A), which is one of the primary flavonoids isolated from the root of the Xinjiang licorice* Glycyrrhiza inflate* [22], has various biological activities, including anti-inflammatory, antioxidant, antitumorigenic, and antimicrobial activities [23, 24]. However, the cytoprotective effect of Lico A against oxidative stress damage has not yet been demonstrated in RAW 264.7 cells. Accordingly, we investigated the cytoprotective effect of Lico A and the mechanism related to oxidative stress in* tert*-butyl hydroperoxide- (*t*-BHP-) induced RAW 264.7 cells.

## 2. Materials and Methods

### 2.1. Reagents and Chemical

Licochalcone A (Lico A), purity >98%, used in the experiments was obtained from the National Institute for the Control of Pharmaceutical and Biological Products (Beijing, China). 3-(4,5-Dimethylthiazol-2-yl)-2,5-diphenyltetrazolium bromide (MTT),* tert*-butyl hydroperoxide (*t*-BHP), dimethyl sulfoxide (DMSO), U0126, SB203580, SP600125, and LY294002 (specific inhibitors of the ERK1/2, p38, JNK1/2, and PI3K/Akt, resp.) were purchased from Sigma-Aldrich (St. Louis, MO). Penicillin and streptomycin, fetal bovine serum (FBS), and Dulbecco's modified Eagle's medium (DMEM) were purchased from Invitrogen-Gibco (Grand Island, NY). Antibodies against Nrf2, HO-1, Keap1, Akt, phospho-Akt, phospho-extracellular signal-regulated kinase (ERK), ERK, phospho-c-Jun NH2-terminal kinase (JNK), JNK, phospho-p38, p38, Lamin B, and *β*-actin were purchased from Cell Signaling (Boston, MA, USA) or Abcam (Cambridge, MA, USA). The horseradish peroxidase- (HRP-) conjugated anti-rabbit or anti-mouse IgG were obtained from Proteintech (Boston, MA, USA). Tin protoporphyrin IX (SnPP IX, HO-1 inhibitor) was purchased from Calbiochem (La Jolla, CA, USA). The control siRNA and Nrf2 siRNA were purchased from Santa Cruz Biotechnology (Santa Cruz, CA, USA). Faststart Universal SYBR Green Master was purchased from Roche (Basel, Switzerland). Prime-Script RT-PCR kit was purchased from Takara (Dalian, China). In addition, GSH test kit was obtained from Nanjing Jiancheng Bioengineering Institute (Nanjing, China).

### 2.2. Cell Culture and Cell Treatment

The RAW 264.7 mouse macrophage cell line, purchased from the China Cell Line Bank (Beijing China), was grown in DMEM medium supplemented with 3 mM glutamine, 10% foetal bovine serum (FBS), and 100 U/mL of penicillin and 100 U/mL of streptomycin at 37°C in a humidified atmosphere containing 5% CO_2_. In all experiments, cells were allowed to acclimate for 24 h before any treatments.

### 2.3. Cell Viability Assay

According to the manufacturer's instructions, cell viability was evaluated by MTT assay. RAW 264.7 cells were seeded in 96-well plates at the concentration of 3 × 10^4^ cells/well. After 24 h, cells were treated with* t*-BHP only, Lico A and* t*-BHP, or Lico A,* t*-BHP, and selective inhibitors at an indicated concentration and time. Then, the cells were added with MTT (5 mg/mL) and incubated for another 4 h, the supernatant was removed, and DMSO was added to each well to lyse the cells. The absorbance of MTT was measured at 570 nm.

### 2.4. Quantification of Apoptotic and Necrosis Cells

RAW 264.7 cells were seeded into 12-well plates (5 × 10^5^ cells/well) for 24 h incubation, and then were exposed to various concentrations of Lico A for 18 h and subjected to* t*-BHP (10 *μ*M) for additional 3 h. Subsequently, cells were washed twice with ice-cold PBS, collected, and centrifuged at 1500 rpm/min for 5 min, 4°C. Next, cells were subjected to Hoechst 33342 and propidium iodide staining and the percentage of apoptosis and necrosis were determined using flow cytometry (LSR II Flow Cytometer; BD Biosciences, San Jose, CA, USA).

### 2.5. Detection of Intracellular ROS Levels

To measure intracellular ROS production, RAW 264.7 cells were grown in 24-well plates (1 × 10^5^ cells/well) for 24 h incubation and then recovered in serum-free DMEM for 6 h; the cells were then preincubated with various concentrations of Lico A for 18 h. Next, the cells were stained with 50 *μ*M of DCFH-DA for 1 h and subsequently incubated with* t*-BHP (10 *μ*M) for 30 min to induce the ROS generation. DCF fluorescence intensities were measured in a multidetection reader (Bio-Tek Instruments Inc.) at an excitation and emission wavelength of 485 nm and 535 nm, respectively.

### 2.6. Measurement of Intracellular GSH Levels

To measure intracellular reduced glutathione (GSH) levels, RAW 264.7 cells were grown in 6-well plates (1 × 10^6^ cells/well) for 24 h incubation, and then the cells were exposed to various concentrations of Lico A for 18 h and subsequently subjected to* t*-BHP (10 *μ*M) for 3 h. According to the manufacturer's instructions, the level of intracellular of GSH was quantified using a commercially available GSH test kit (Nanjing Jiancheng Bioengineering Institute, Nanjing, China). The absorbance was measured at 405 nm using a microplate reader (Bio-Tek Instruments Inc.).

### 2.7. Total RNA Extraction and qPCR

Total RNA from cells was isolated using Trizol reagent according to the procedure described by the manufacturer. After the concentration of RNA was determined by spectrophotometer, 1 *μ*g of RNA was transformed into cDNA using Prime-Script RT-PCR kit (Takara). The following PCR primer sequences (forward and reverse, resp.) were used: GCLC: 5′-ACG GCT GCT ACG ACA ACG GCC CTC-3′ and 5′-ACC CAG CGG TGC AAA CTC CGC GC-3′; GCLM: 5′-TCC TCT CGA AGA GGG CGT GTC CAG-3′ and 5′-AGG GAG G GA AGG AAG GGA GGG AG-3′; *β*-actin: 5′-TCT GTG TGG ATT GTG GCT CTA-3′ and 5′-CTG CTT GCT GAT CCA CAT CTG-3′. PCR reactions were carried out using the SYBR green working solution and quantitatively measured with the Applied Biosystems 7300 real-time PCR system and software (Applied Biosystems, Carlsbad, CA, USA). The following thermal cycler parameters were used: 95°C for 10 min, followed by 40 cycles of 95°C for 10 s, and 60°C for 30 s. Gene expression changes were calculated by the comparative Ct method and the values were analyzed by normalizing with *β*-actin mRNA expression.

### 2.8. Western Blot Analysis

The RAW 264.7 mouse macrophage cell line (1 × 10^6^ cells/well in 6-well plate) was washed twice with ice-cold PBS, collected, and centrifuged at 6000 rpm/min for 5 min, 4°C. Then, the cells were lysed in a RIPA with protease and phosphatase inhibitors for 30 min. The protein concentrations were measured using a BCA protein assay kit (Beyotime, China). An equal amount of protein (40 *μ*g) for each sample was resolved by sodium dodecyl sulfate-polyacrylamide gel electrophoresis (SDS-PAGE) using a 10% gel and then electrophoretically transferred onto a polyvinylidene difluoride membranes (PVDF), which was purchased from Bio-Rad (Hercules, CA). The membrane was blocked with blocking solution (5% (w/v) nonfat dry milk) for 2 h and followed by an overnight incubation at 4°C with specific primary antibody, including Keap1, Nrf2, HO-1, p-Akt/Akt, p-JNK/JNK, p-ERK/ERK, p-p38/p38, Lamin B, and *β*-actin. Next day, after thoroughly washing with TBST for three times, the membrane was incubated for an additional 2 h with a peroxidase conjugated secondary antibody at room temperature and followed by ECL detection (Millipore corporation, Billerca, MA, USA). *β*-actin and Lamin B were used as loading controls for whole, cytosolic, and nuclear cell proteins, respectively. Band intensities were quantified by using ImageJ gel analysis software. The experiments were repeated three times for each experimental condition.

### 2.9. Preparation of Nuclear and Cytosolic Fractions

The nuclear and the cytoplasmic extracts were prepared using an NE-PER Nuclear and Cytoplasmic Extraction Reagents kit (Pierce Biotechnology, Rockford, IL, USA), in accordance with the manufacturer's instructions. All steps were carried out on ice or at 4°C unless stated otherwise.

### 2.10. Nrf2-siRNA Transfection

For Nrf2-siRNA transfection, RAW 264.7 cells were grown in 6-well plates (2 × 10^5^ cells/well) until the confluence of cells reached approximately 50%. Then, Nrf2-siRNA or Nrf2-negative control siRNA was transiently transfected into the cells using siRNA transfection reagent lipofectamine 2000 in accordance with the manufacturer's protocol (Santa Cruz Biotechnology, Santa Cruz, CA). After 6 h, the transfected cells were treated with Lico A for 18 h and followed by lysis buffer for Western blot analysis.

### 2.11. ARE Promoter Activity

RAW 264.7 cells were grown in 24-well plates (2 × 10^5^ cells/well) until the confluence of cells reached approximately 50%. According to the manufacturer's protocol of Invitrogen (Carlsbad, CA, USA), pRL-TK and pGL-ARE plasmids were transfected into cells using Lipofectamine 2000. After Lico A (3.7 *μ*M) treatment for different periods, we used a dual-luciferase reporter assay system (Dual-Glo Luciferase Assay System) for detecting and analyzing ARE-driven promoter activity.

### 2.12. Statistical Analysis

All results were expressed as means ± SEM of three independent experiments. Differences between mean values of normally distributed data were analyzed using two-tailed Student's* t*-test. Statistical significance was accepted when *P* < 0.05 or *P* < 0.01.

## 3. Results

### 3.1. Lico A Protected against* t*-BHP-Induced Cytotoxicity and Reduced Apoptosis Percentage in RAW 264.7 Cells

**Figure 1:**
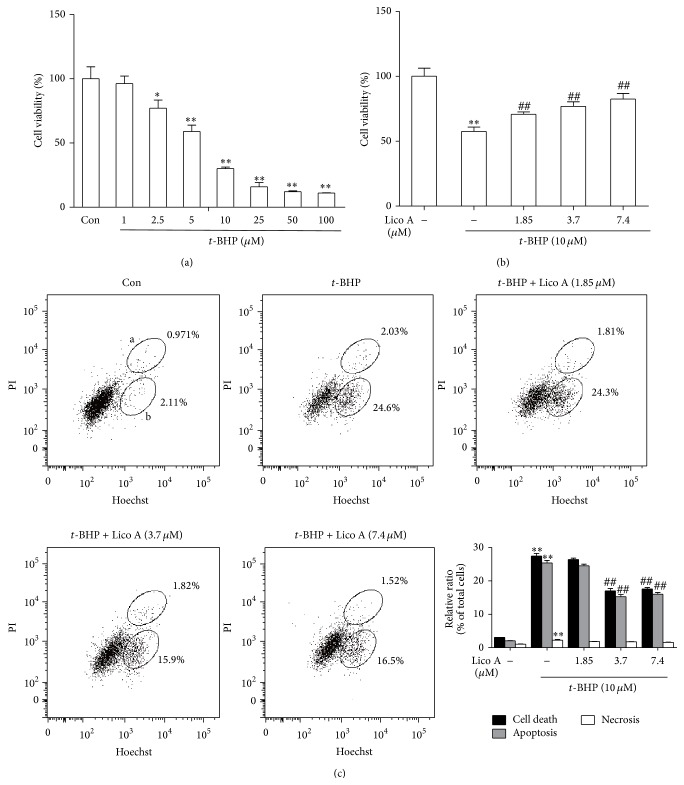
Effects of Lico A on* t*-BHP-induced RAW 264.7 cell cytotoxicity, apoptosis, and necrosis. (a) RAW 264.7 cells were treated by 0, 1, 2.5, 10, 25, 50, and 100 *μ*M* t*-BHP for 24 h. (b) Cells were pretreated with Lico A (1.85, 3.7 and 7.4 *μ*M) for 18 h, subsequently exposed to* t*-BHP (10 *μ*M) for 3 h. Cell viability after* t*-BHP exposure was measured by MTT assay. (c) Cells were exposed to various concentrations of Lico A for 18 h and subsequently subjected to* t*-BHP (10 *μ*M) for 3 h. The percentage of cell apoptosis and necrosis were determined using flow cytometry. (a and b) represent necrosis and apoptosis, respectively. All results were expressed as means ± SEM of three independent experiments. ^*^ *P* < 0.05, ^**^ *P* < 0.01 versus the control group; ^#^ *P* < 0.05, ^##^ *P* < 0.01 versus the *t*-BHP group.

*t*-BHP is commonly used to induce oxidative stress in biological systems. The viability of RAW 264.7 cells decreased in a dose-dependent manner after 24 h of incubation with* t*-BHP. However, a significant difference between the control and* t*-BHP-stimulated group was observed only at 10 *μ*M* t*-BHP (Figure 1(a)). Therefore, we chose 10 *μ*M* t*-BHP as the treatment dose to induce obvious oxidative injury. Moreover, the RAW 264.7 cells were pretreated with various concentrations of Lico A (1.85, 3.7 and 7.4 *μ*M) for 18 h and subsequently exposed to* t*-BHP (10 *μ*M) for 3 h to investigate the protective effects of Lico A on these cells. Our results suggested that Lico A significantly protected these cells against* t*-BHP-induced oxidative cytotoxicity (Figure 1(b)). Furthermore,* t*-BHP also markedly induced cell death through increasing the percentage of apoptosis and necrosis in total cells, and Lico A effectively decreased* t*-BHP-induced cell apoptosis at concentrations of 1.85, 3.7, and 7.4 *μ*M, whereas Lico A could not attenuate* t*-BHP-induced necrosis cells (Figure 1(c)).

### 3.2. Lico A Inhibited* t*-BHP-Induced ROS Production and GSH Depletion and Enhanced GCLC and GCLM Expression in RAW 264.7 Cells

**Figure 2:**
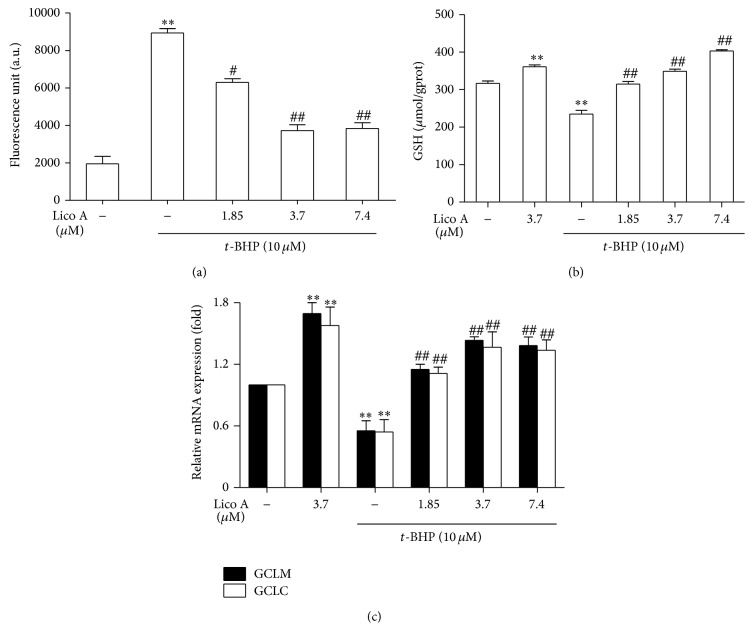
Effects of Lico A on* t*-BHP-induced ROS generation, GSH levels, and GCLM and GCLC genes expression in RAW 264.7 cells. RAW 264.7 cells were pretreated with or without Lico A for 18 h and then were exposed to* t*-BHP for additional 3 h. (a) Effect of Lico A on* t*-BHP-induced ROS generation in RAW 264.7 cells. The ROS generation was determined in accordance with the Experimental Section. (b) Effect of Lico A on* t*-BHP-induced GSH depletion was determined using a commercial GSH test kit. (c) Effects of Lico A on GCLM and GCLC genes expression. Total RNA was extracted from RAW 264.7 cells and genes expression was quantified using real-time PCR. All results were expressed as means ± SEM of three independent experiments. ^*^ *P* < 0.05, ^**^ *P* < 0.01 versus the control group; ^#^ *P* < 0.05, ^##^ *P* < 0.01 versus the* t*-BHP group.

Due to* t*-BHP elevating ROS generation to induce cell oxidative damage, we investigated that Lico A protected against* t*-BHP-induced oxidative injury via inhibiting intracellular ROS production in RAW 264.7 cells. In this study,* t*-BHP treatment markedly increased ROS production, which was inhibited by Lico A treatment (Figure 2(a)). In addition, GSH is recognized to be a vital antioxidant, which protects against* t*-BHP-induced oxidative injury. In fact, GCLC and GCLM are closely associated with the expression of GSH level. Hence, we examined GSH contents as well as GCLC and GCLM expression in* t*-BHP-exposed cells pretreated with or without Lico A. Our results showed that* t*-BHP treatment considerably enhanced GSH depletion, whereas Lico A treatment significantly decreased the depletion of GSH* t*-BHP-induced and increased the expression of GCLC and GCLM (Figures 2(b) and 2(c)).

### 3.3. Lico A Upregulated HO-1 Protein Expression in RAW 264.7 Cells

**Figure 3:**
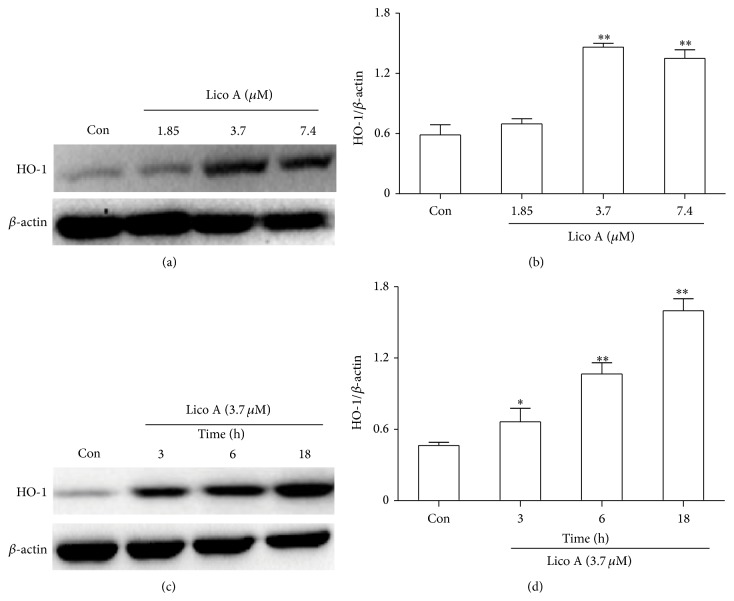
Effects of Lico A on HO-1 protein expression in RAW 264.7 cells. (a) Cells were treated with increasing doses of Lico A (1.85, 3.7, and 7.4 *μ*M) for 18 h, and (c) cells were treated with Lico A (3.7 *μ*M) indicated time periods. Protein expression of HO-1 was determined by Western blot analysis. (b and d) Quantification of HO-1 protein expression was performed by densitometric analysis and *β*-actin acted as an internal control. All results were expressed as means ± SEM of three independent experiments. ^*^ *P* < 0.05, ^**^ *P* < 0.01 versus the control group.

Because HO-1 is an essential component of the cellular defense against oxidative stress, we examined whether Lico A-induced HO-1 expression increased the resistance of RAW 264.7 cells to oxidative injury. RAW 264.7 cells were treated with Lico A for 18 h to determine the most effective concentration for increasing HO-1 protein expression (Figures 3(a) and 3(b)). The cells were treated with Lico A (3.7 *μ*M) for different periods to determine the optimal exposure period for enhancing HO-1 protein expression (Figures 3(c) and 3(d)). Our results showed that exposure to 3.7 *μ*M Lico A for 18 h dramatically upregulated HO-1 protein expression in RAW 264.7 cells.

### 3.4. Lico A Enhanced Nrf2 Protein Expression and ARE Activation and Increased Keap1 Degradation in RAW 264.7 Cells

**Figure 4:**
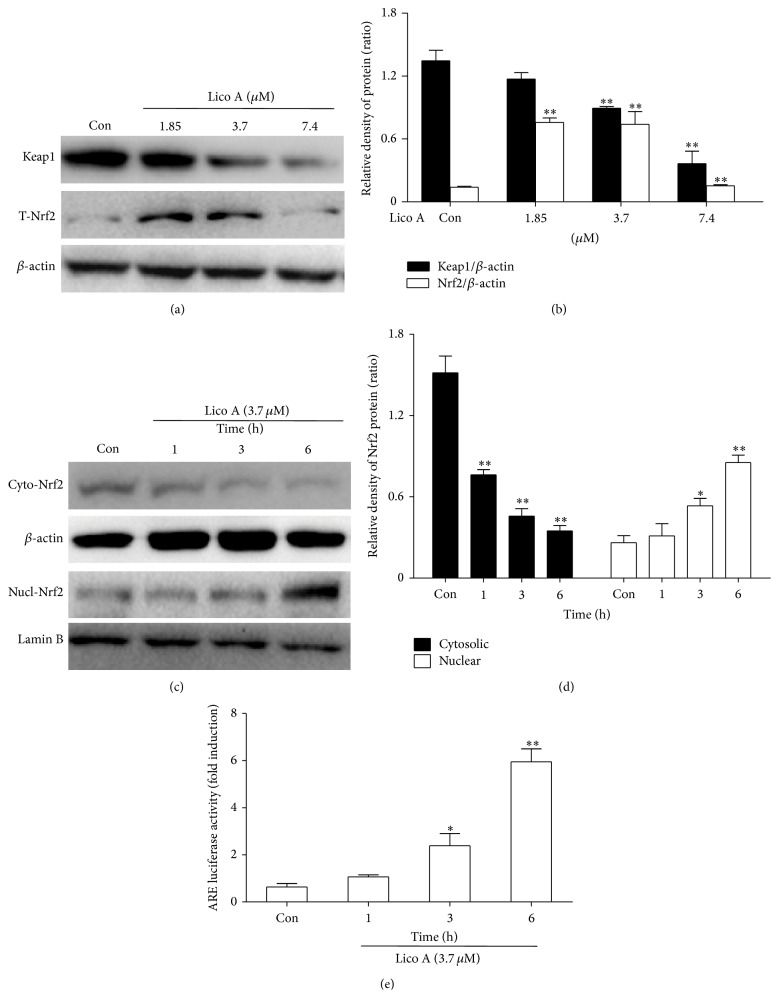
Effects of Lico A on the Keap1/Nrf2/ARE signaling pathway in RAW264.7 cells. (a) Cells were treated with different concentration of Lico A (1.85, 3.7 and 7.4 *μ*M) for 18 h, and the total protein were measured by Western blot analysis. (c) Cells were treated with Lico A (3.7 *μ*M) indicated time periods, and the nuclear and cytoplasmic levels of Nrf2 were examined by Western blot analysis. (b and d) The relative density of protein was performed by densitometric analysis; *β*-actin and Lamin B acted as an internal control, respectively. (e) The luciferase plasmids pGL-ARE and pRL-TK was transiently transfected into cells for 24 h and subsequently exposed to 3.7 *μ*M Lico A for the indicated periods. ARE luciferase activity was detected by a dual-luciferase reporter assay system. All results were expressed as means ± SEM of three independent experiments. ^*^ *P* < 0.05, ^**^ *P* < 0.01 versus the control group.

Nrf2 regulates the antioxidant responses via transcriptionally activating the HO-1 gene expression. In addition, Keap1 negatively regulates Nrf2 through inhibiting the Nrf2 activation. Consequently, we examined whether Lico A could induce Nrf2 activation and Keap1 degradation in association with HO-1 upregulation. RAW 264.7 cells were treated with Lico A (1.85, 3.7 and 7.4 *μ*M) for 18 h, and then total protein was extracted from the cells for Western blot analysis. The results showed that 3.7 *μ*M Lico A significantly increased the total protein expression of Nrf2 and the degradation of Keap1 (Figures 4(a) and 4(b)). Thus, we furthermore examined whether 3.7 *μ*M Lico A could lead to a decrease in the cytoplasmic levels and a concomitant increase in the nuclear levels of Nrf2 in a time-dependent manner (Figures 4(c) and 4(d)). In addition, due to the increased Nrf2 expression in the nucleus is required for ARE activation, the ARE-luciferase plasmid was transiently transfected into the cells and then were exposed to Lico A, as well as changes in luciferase activity were used as a measure of ARE activation. The results of this assay suggested that Lico A also markedly increased ARE-driven luciferase activity in a time-dependent manner (Figure 4(e)).

### 3.5. Lico A Increased Nrf2-Mediated HO-1 Protein Expression in RAW 264.7 Cells

**Figure 5:**
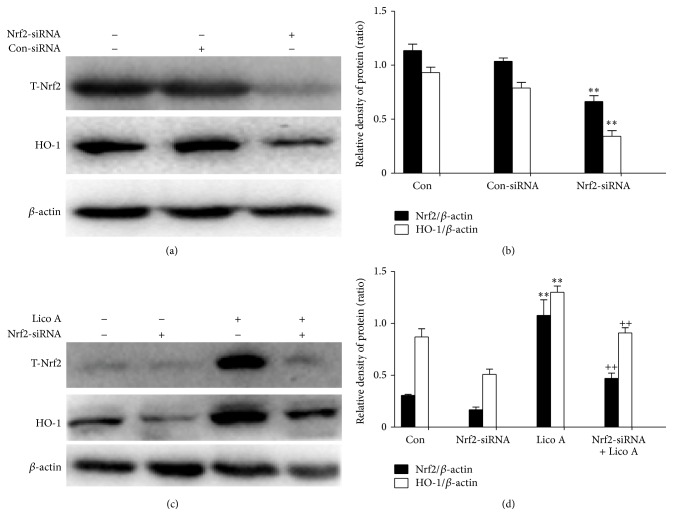
Effects of Nrf2-siRNA transfection on Lico A-induced HO-1 protein expression in RAW 264.7 cells. (a) Nrf2-siRNA or Nrf2-negative control siRNA was transfected into cells for 24 h and was collected; proteins were detected by Western blot analysis. (c) Nrf2 mediates Lico A-induced HO-1 protein expression. Nrf2-siRNA or Nrf2-negative control siRNA were transfected into cells for 6 h and then were treated with Lico A (3.7 *μ*M) for 18 h. (b and d) The relative density of protein was performed by densitometric analysis and *β*-actin acted as an internal control. All results were expressed as means ± SEM of three independent experiments. ^**^ *P* < 0.01 versus the control group; ^++^ *P* < 0.01 versus the Lico A group.

Several previous reports showed that Nrf2 is essential for HO-1 regulation. Accordingly, we attempted to investigate whether the upregulation of HO-1 expression is mediated by Nrf2. The role of Nrf2 in Lico A-induced HO-1 expression was confirmed using siRNA to knockdown Nrf2. Control or Nrf2 siRNA was transiently transfected into RAW 264.7 cells, and then Nrf2 and HO-1 protein expression was measured by Western blot analysis. The data demonstrated that Nrf2 siRNA markedly inhibited total Nrf2 and HO-1 protein expression to a similar extent when compared with the negative control (Figures 5(a) and 5(b)). Additionally, to further investigate whether Lico A-induced HO-1 protein expression is mediated by Nrf2, we measured HO-1 expression after Nrf2 siRNA transfection. Our studies indicated that Lico A-increased HO-1 protein expression significantly decreased in Nrf2 siRNA-transfected cells, whereas the same amount of nonspecific control siRNA did not affect HO-1 expression in RAW 264.7 cells (Figures 5(c) and 5(d)). These results further provided a support that the upregulation of HO-1 expression is mediated primarily through the transcriptional activator Nrf2.

### 3.6. Lico A Activated the PI3K/Akt and MAPK Pathways in RAW 264.7 Cells

**Figure 6:**
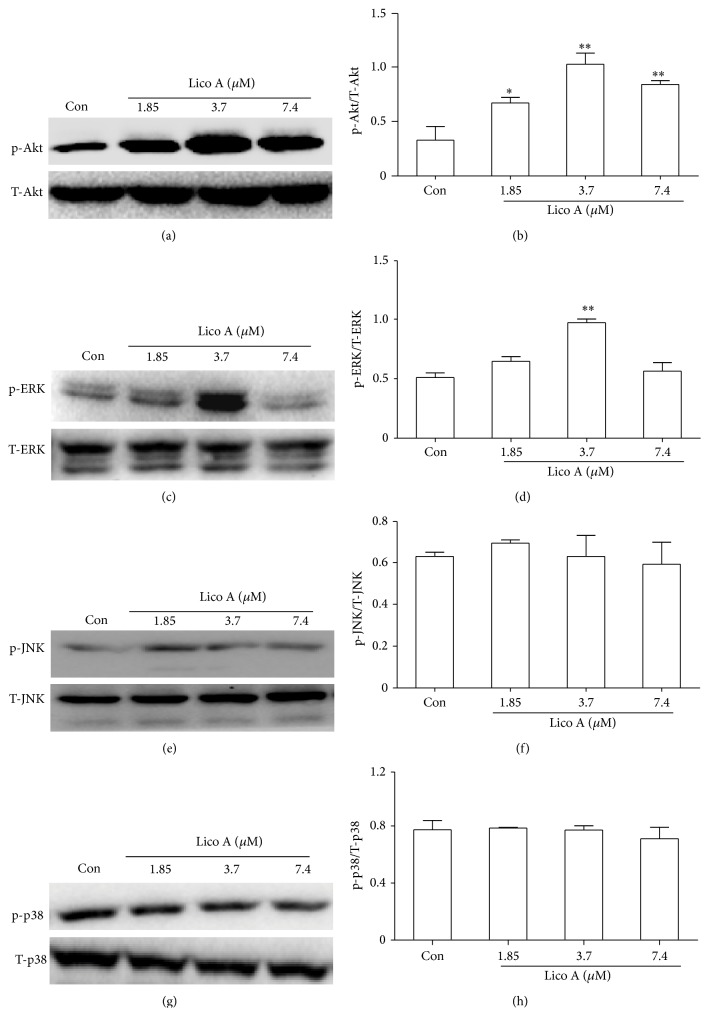
Effects of Lico A on the activation of the PI3K/Akt and MAPK pathways in RAW 264.7 cells. Cells were treated with increasing doses of Lico A (1.85, 3.7 and 7.4 *μ*M) for 18 h, and whole cell lysates were prepared and detected by Western blot analysis for phosphorylated and total Akt, ERK, JNK, and p38 protein expression. (b, d, f, and h) Quantification of induction of PI3K/Akt and MAPKs phosphorylation were performed by densitometric analysis and its unphosphorylated forms acted as an internal control, respectively. All results were expressed as means ± SEM of three independent experiments. ^*^ *P* < 0.05, ^**^ *P* < 0.01 versus the control group.

Recent reports have suggested that several signal transduction pathways, such as the PI3K and MAPK pathways are involved in the regulation of Nrf2 nuclear translocation. ERK and c-Jun N-terminal kinase (JNK) positively regulate the Nrf2 pathway whereas p38 MAPK exerts both positive and negative regulations [8, 25]. They all belong to members of MAPK family. Therefore, we examined the activation of PI3K/Akt and MAPK pathway in RAW 264.7 cells. RAW 264.7 cells were exposed to Lico A (1.85, 3.7, and 7.4 *μ*M) for 18 h, and then total protein was extracted from these cells for Western blot analysis using specific antibodies. The results indicated that 3.7 *μ*M Lico A clearly increased Akt and ERK phosphorylation in RAW 264.7 cells. In contrast, the phosphorylation of p38 and JNK was not activated at these three concentrations of Lico A (Figure 6).

### 3.7. Lico A Modulated HO-1 Expression and Nrf2 Nuclear Translocation via Akt and ERK Activation in RAW 264.7 Cells

**Figure 7:**
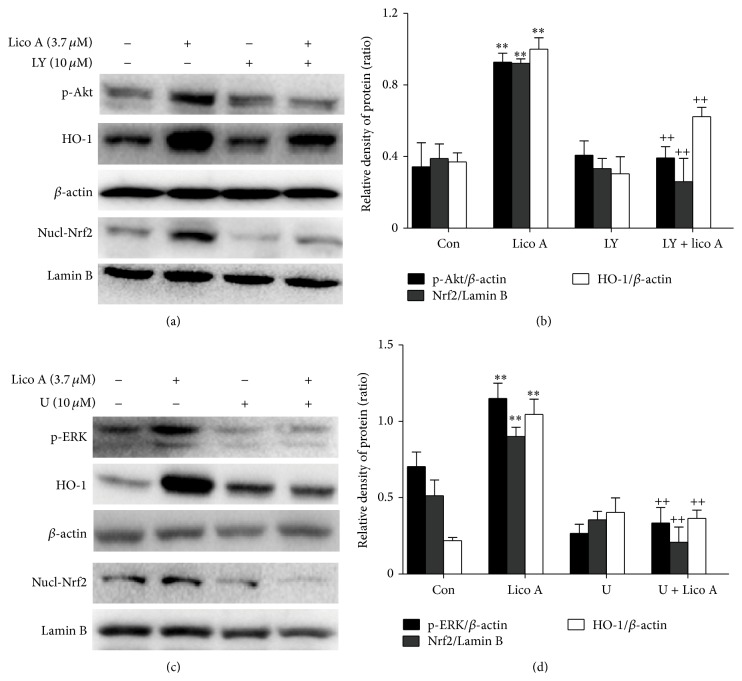
Effects of Lico A-induced Akt and ERK activation on HO-1 expression and Nrf2 nuclear translocation. Cells were pretreated with LY294002 (10 *μ*M) and U0126 (10 *μ*M) for 6 h and then were exposed to Lico A (3.7 *μ*M) for 18 h. The whole cells lysates were examined by Western blot analysis with anti-HO-1 and anti-*β*-actin antibodies, and nuclear extracts were subjected to Western blot analysis with anti-Nrf2 and anti-Lamin B antibodies. (b and d) The relative density of protein was performed by densitometric analysis; *β*-actin and Lamin B acted as an internal control, respectively. All results were expressed as means ± SEM of three independent experiments. ^**^ *P* < 0.01 versus the control group;^ ++^ *P* < 0.01 versus the Lico A group.

To further determine the upstream signaling pathway involved in Lico A-mediated Nrf2 activation and HO-1 induction, we investigated the effects of LY294002 and U0126, which are specific inhibitors of the PI3K/Akt and ERK pathways, respectively, on Nrf2 nuclear translocation and HO-1 protein expression. RAW 264.7 cells were treated with either LY294002 (20 *μ*M) or U0126 (10 *μ*M) for 6 h and then exposed to Lico A (3.7 *μ*M) for 18 h. We found that Lico A-mediated Nrf2 activation and HO-1 induction were dramatically inhibited by PI3K/Akt and ERK kinase inhibitors, respectively (Figure 7). These investigations suggested that Lico A modulated Nrf2 nuclear translocation and HO-1 expression via the activation of PI3K/Akt and ERK signaling in RAW 264.7 cells.

### 3.8. Lico A Alleviated Cellular Injury by Upregulating Nrf2 and HO-1 via Akt and ERK Activation in RAW 264.7 Cells

**Figure 8:**
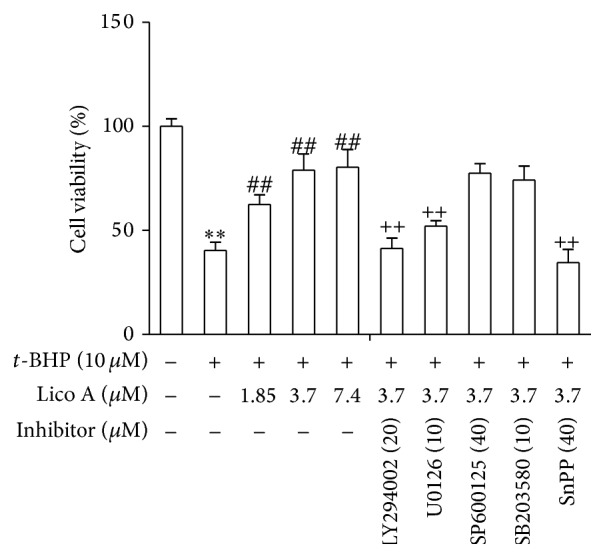
Effects of Lico A-induced Akt and ERK pathway activation on* t*-BHP-induced cytotoxicity in RAW 264.7 cells. Cells were pretreated with or without LY294002 (20 *μ*M), U0126 (10 *μ*M), SB203580 (10 *μ*M), SP600125 (40 *μ*M), and SnPP (40 *μ*M), respectively, for 6 h and treated with Lico A for 18 h. Then, cells were exposed to* t*-BHP (10 *μ*M) for 10 h. Cell viability after* t*-BHP exposure was measured by MTT assay. All results were expressed as means ± SEM of three independent experiments. ^**^ *P* < 0.01 versus the control group; ^##^ *P* < 0.01 versus the* t*-BHP group; ^++^ *P* < 0.01 versus the Lico A (3.7 *μ*M) +* t*-BHP group.

Based on the above outcomes, we hypothesized that the protective effects of Lico A against* t*-BHP-induced oxidative stress result from the induction of antioxidant genes, such as HO-1 and its transcription factor Nrf2. Furthermore, we hypothesized that the PI3K/Akt and MAPK pathways, which are upstream signaling pathways, are involved in Lico A-mediated Nrf2 activation and HO-1 induction. Thus, RAW 264.7 cells were pretreated with LY294002 (PI3K/Akt inhibitor, 20 *μ*M), U0126 (ERK inhibitor, 10 *μ*M), SB203580 (p38 inhibitor, 10 *μ*M), SP600125 (JNK inhibitor, 40 *μ*M), or SnPP (HO-1 inhibitor, 40 *μ*M) for 6 h, respectively, and then treated with Lico A (3.7 *μ*M) for 18 h. Next, the cells were exposed to 10 *μ*M* t*-BHP for 10 h to determine cell viability. Our results suggested that Lico A pretreatment significantly increased cell viability compared with that of* t*-BHP-treated cells. In contrast, this effect was partially inhibited in the presence of ERK, PI3K/Akt, and HO-1 inhibitors, whereas JNK and p38 inhibitors could not inhibit this effect (Figure 8). This result showed that Lico A induced HO-1 expression via the activation of Akt, ERK, and Keap1/Nrf2/ARE signaling pathways in RAW 264.7 cells.

## 4. Discussion and Conclusion

Excessive exposure to reactive oxygen species (ROS) causes oxidative stress. Additionally, excessive ROS production inflicts damage on essential cellular macromolecules including lipids, proteins, and DNA; this damage results in several human diseases, such as inflammation, cancer, atherosclerosis, rheumatoid arthritis, and neurodegenerative diseases [26]. Therefore, ROS clearance and oxidative stress inhibition may play essential roles in preventing numerous diseases. Various natural products, particularly flavonoids, possess multiple cytoprotective effects through free radical scavenging activity [27]. Lico A, which is a flavonoid, possesses radical-scavenging and antioxidant effects [24]. However, the mechanism underlying the biological effects of Lico A in RAW 264.7 cells remains unclear. Our present study aimed to investigate whether Lico A has the ability to induce GSH, GCLC, and GCLM enhancement, to modulate HO-1 induction and Nrf2 nuclear translocation and to protect against* t*-BHP-induced oxidative damage and cell death via ERK and Akt activation in RAW 264.7 cells.

Previous reports suggested that* t*-BHP exposure could not only lead to cell death via inducing apoptosis but also result in oxidative stress via increasing ROS formation. ROS are associated with cell damage and with chronic disease development [28, 29]. Additionally, it is reported that the overproduction of GCL, which comprised of GCLC and GCLM, enhances total GSH contents and protects against H_2_O_2_-induced cell death in human granulose tumour cells [30]. On the other hand, GSH, which is a nonenzymatic antioxidant, cofactor, or coenzyme, plays an essential role in directly involving in the production and clearance of ROS [31]. For example, previous reports showed that antcin C reduced the depletion of GSH levels in the* t*-BHP-exposed HepG2 cells and mice liver tissues [32]. Hence, the aim of this study was to evaluate the ability of the antioxidant Lico A to reduce oxidant-induced cellular damage in RAW 264.7 cells. Our experimental results showed that* t*-BHP-induced RAW 264.7 cells displayed significantly decreased cell viability in a dose-dependent manner; the viability of RAW 264.7 cells treated with 10 *μ*M* t*-BHP decreased up to 36.72% compared with the control group (Figure 1(a)). However, Lico A pretreatment markedly enhanced cell viability and inhibited* t*-BHP-induced cell apoptosis (Figures 1(b) and 1(c)). Furthermore, increased ROS production and decreased GSH levels are closely associated with apoptosis [33]. In present study, we found that Lico A significantly reduced ROS formation and GSH depletion as well as enhanced GCLC and GCLM genes expression in the* t*-BHP-stimulated RAW 264.7 cells (Figure 2).

Increasing evidence suggests that the cytoprotective properties of antioxidants are generally related to their ability to induce antioxidative enzymes, such as HO-1. HO-1, which is an enzyme that is essential for heme degradation, has been recognized as an important cellular defense mechanism against various stresses, including oxidative stress [11, 34]. Our results indicated that different concentrations and exposure periods of Lico A treatment markedly increased HO-1 induction in RAW 264.7 cells (Figure 3). Furthermore, Keap1/Nrf2/ARE signaling plays a crucial role in protecting cells against endogenous and exogenous stresses [35]. The transcriptional activation of Nrf2 is dependent on the rate of nuclear translocation, followed by the disassociation of Nrf2 from cytoplasmic Keap1, which leads to the induction of some cytoprotective proteins, including HO-1, GCLC, and GCLM [36, 37]. In this study, we found that Lico A treatment increased Nrf2 protein expression and decreased Keap1 protein expression in total cell lysates (Figures 4(a) and 4(b)). In addition, Lico A treatment markedly promoted the nuclear translocation of Nrf2, which was directly proportional to the decrease in Nrf2 in the cytoplasm (Figures 4(c) and 4(d)). Nrf2 is released from Keap1 and is translocated to the nucleus, where Nrf2 binds to ARE in the promoter region of its target genes, thereby inducing many cytoprotective genes and antioxidative enzymes [38]. As shown in Figure 4(e), Lico A treatment significantly enhanced ARE luciferase activity in a time-dependent manner, which indicated the inducing ability of Lico A on various ARE-regulated genes, such as HO-1. However, transient transfection with Nrf2 siRNA partially abolished Lico A-induced HO-1 expression (Figure 5), which suggested that Lico A induced HO-1 protein expression via the Keap1/Nrf2/ARE signaling pathway in RAW 264.7 cells.

Many previous reports have suggested that the PI3K/Akt and MAPK pathways play a key role in regulating HO-1 expression and Nrf2-dependent transcription [39, 40]. The aim of the present experiment was to investigate a possible role, Lico A-induced HO-1 expression which was the activation of the PI3K/Akt and MAPK pathways. Our results showed that Lico A induced HO-1 expression via activating the PI3K/Akt and ERK pathways, whereas JNK and p38 MAPK signaling molecules did not affect HO-1 expression (Figure 6). Moreover, MAPK and PI3K/Akt are candidate upstream signaling pathways for Nrf2-related HO-1 regulation in RAW 264.7 cells [41]. This study used specific inhibitors of the PI3K/Akt and ERK pathways to further investigate whether the activation of the PI3K/Akt and ERK MAPK signaling pathways was a required event for HO-1 expression and Nrf2 nuclear translocation. As shown in Figure 7, the addition of U0126 and LY294002 significantly abolished Lico A-induced HO-1 protein expression and Nrf2 nuclear translocation. These results indicated that the PI3K/Akt and ERK pathways are important for Lico A-induced HO-1 expression and Nrf2 nuclear translocation. Furthermore, cell viability decreased significantly when Lico A treatment was combined with PI3K/Akt, ERK, and HO-1 inhibitors in* t*-BHP-induced RAW 264.7 cells (Figure 8). These results suggested that Lico A induced HO-1 expression via the activation of Akt, ERK, and Keap1/Nrf2/ARE signaling in RAW 264.7 cells.

In conclusion, the present study demonstrated that Lico A could protect RAW 264.7 cells via the suppression of* t*-BHP-induced oxidative damage, apoptosis, ROS production, and GSH depletion as well as the enhancement of GCLC and GCLM genes expression. Furthermore, Lico A could not only induce Nrf2 nuclear translocation, which is upstream of Lico A-induced antioxidant gene expression, but also activate Akt and ERK phosphorylation. Moreover, the PI3K/Akt and ERK pathways are associated with Lico A-induced Nrf2 nuclear translocation, HO-1 expression, and cytoprotection. This study provides biological evidence supporting the application of Lico A in the treatment of oxidative stress-induced disorders.
